# Ciprofloxacin for the Treatment of Infections Caused by Carbapenemase-Producing Gram-Negative Bacteria †

**DOI:** 10.3390/antibiotics13121138

**Published:** 2024-11-26

**Authors:** Pablo Rubiñan, Belén Viñado, Nuria Fernández-Hidalgo, Nieves Larrosa, Abiu Sempere, David Campany, Dolors Rodríguez-Pardo, Juan José González-López, Xavier Nuvials, Ester del Barrio-Tofiño, Laura Escolà-Vergé, Ibai Los-Arcos

**Affiliations:** 1 Infectious Diseases Unit, Internal Medicine Department, Complexo Hospitalario Universitario de Vigo, 36312 Vigo, Spain; pablo.rubinan.iglesias@sergas.es; 2 Microbiology Department, Hospital Universitari Vall d’Hebron, 08035 Barcelona, Spain; belen.vinado@vallhebron.cat (B.V.); nieves.larrosa@vallhebron.cat (N.L.); juanjo.gonzalez@vallhebron.cat (J.J.G.-L.); ester.delbarrio@vallhebron.cat (E.d.B.-T.); 3 Department of Medicine, Universitat Autònoma de Barcelona, 08035 Barcelona, Spain; nuria.fernandezhidalgo@vallhebron.cat (N.F.-H.); dolors.rodriguez@vallhebron.cat (D.R.-P.); xavier.nuvials@vallhebron.cat (X.N.); 4 CIBERINFEC, ISCIII—CIBER de Enfermedades Infecciosas, Instituto de Salud Carlos III, 28029 Madrid, Spain; 5 Infectious Diseases Department, Hospital Universitari Vall d’Hebron, 08035 Barcelona, Spain; 6 Infectious Diseases Department, Hospital Clinic de Barcelona, 08036 Barcelona, Spain; sempere@clinic.cat; 7 Pharmacy Department, Hospital Universitari Vall d’Hebron, 08035 Barcelona, Spain; david.campany@vallhebron.cat; 8 Critical Care Department, Hospital Universitari Vall d’Hebron, 08035 Barcelona, Spain; 9 Sepsis Organ Dysfunction and Resuscitation (SODIR) Research Group, Vall d’Hebron Institut de Recerca (VHIR), 08035 Barcelona, Spain; 10 Infectious Diseases Unit, Internal Medicine Department, Hospital de la Santa Creu i Sant Pau, Institut de Recerca Sant Pau, 08025 Barcelona, Spain

**Keywords:** fluoroquinolones, OXA, VIM, multidrug-resistant, carbapenem-resistant

## Abstract

**Background:** There is no experience with ciprofloxacin for the treatment of carbapenemase-producing Gram-negative bacteria (CP-GNB) infections. **Methods:** This is a retrospective single-centre study where we describe the clinical evolution of all consecutive adult patients who received ciprofloxacin monotherapy for the treatment of CP-GNB infections. Primary outcomes were clinical failure (defined as death, lack of clinical improvement or a switch to another drug) at day 14 and 30-day all-cause mortality. **Results:** Nineteen patients were included. Fifteen (79%) were men, the median age was 74 years (IQR 66–79) and the median Charlson comorbidity index was five (IQR 3–6.5). The most frequent infections were: nine complicated urinary tract infections, three soft tissue infections and three intra-abdominal infections. Twenty CP-GNBs were isolated (one patient had a coinfection): nine VIM-type-producing *Enterobacterales*, nine OXA-48-type-producing *Enterobacterales* and two VIM-type-producing *Pseudomonas aeruginosa*. Six (32%) patients had positive blood cultures, and one presented with septic shock. The median duration of ciprofloxacin treatment was 14 days (IQR 10–15). One patient presented with clinical failure at day 14. There was no 30-day mortality. Two patients exhibited microbiological recurrence at day 90. There were no reported adverse effects. **Conclusions:** Monotherapy with ciprofloxacin may be an alternative treatment for selected, clinically stable patients with ciprofloxacin-susceptible CP-GNB infections.

## 1. Introduction

Carbapenemase-producing Gram-negative bacteria (CP-GNB) have emerged as a global health problem. These bacteria are a major concern regarding antimicrobial resistance and have been identified by the World Health Organisation as one of the top global public health and development threats, mainly due to their increasing prevalence and prognosis [1]. As a result of their frequency, some classes of carbapenemases are endemic in some regions of the world [2,3]. Infections caused by CP-GNB are typically healthcare-associated and are related to increased costs, length of hospital stay and mortality [4]. The main risk factors for CP-GNB infection are broad-spectrum antibiotics, prior colonisation, ICU stay and invasive procedures [5]. The optimal treatment for these infections is not well defined [6]. The Infectious Diseases Society of America (IDSA) 2024 guidelines recommend meropenem-vaborbactam, imipenem-cilastatin-relebactam, ceftazidime-avibactam, alone or in combination with aztreonam, or cefiderocol as the first choice depending on the type of carbapenemase produced [7]. In pyelonephritis and complicated urinary tract infections (UTIs), fluoroquinolones and trimethoprim-sulfamethoxazole are also recommended. In the case of fluoroquinolones, the evidence is based on infections caused by non-CP-GNB, as data on ciprofloxacin treatment for CP-GNB infections have not been reported [7]. However, the European Society of Clinical Microbiology and Infectious Diseases (ESCMID) guidelines do not mention the possibility of using fluoroquinolones for treating infections caused by CP-GNB [7,8]. The development of emergence resistance to the newer antibiotics is already a concern [9,10,11]. Sparing use of these new antimicrobial agents and the use of oral sequential therapy could be beneficial for ciprofloxacin treatment. In fact, fluoroquinolones are among the recommended antibiotic treatments for non-severe infections caused by ESBL-producing GNB according to both clinical guidelines [3,4]. In our centre, ciprofloxacin has been used in selected patients who have an infection caused by CP-GNB and who are susceptible to this antimicrobial agent. This study aimed to describe the clinical characteristics and outcomes of patients with infections caused by CP-GNB treated with ciprofloxacin.

## 2. Results

**Figure 1:**
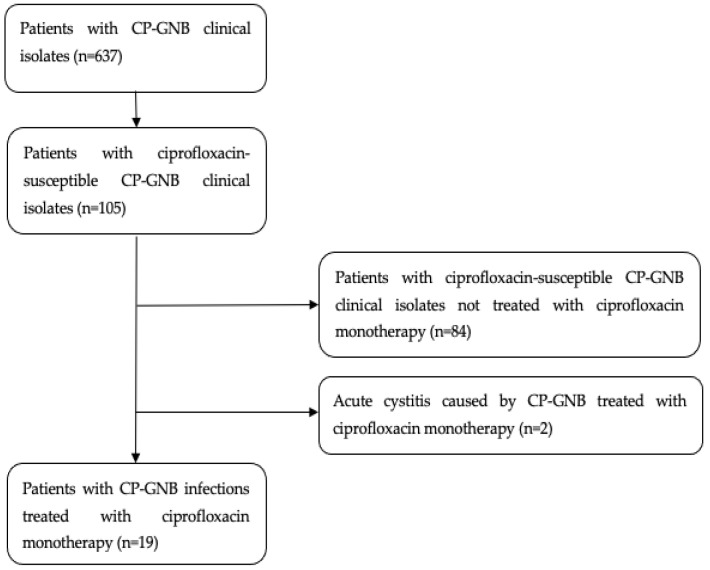
Flow diagram of the patients included in this study.

During the study period, 637 patients with CP-GNB clinical isolates were identified. Of these patients, 105 (16.5%) had isolates susceptible to ciprofloxacin; ultimately, 19 met the study inclusion criteria (Figure 1).

The 19 included patients had a median age of 74 years (interquartile range [IQR] 66–79 years), and 15 (79%) were men. The median Charlson comorbidity index was five (IQR 3–6.5). Eight (42%) patients were immunosuppressed, and four (21%) of them were kidney transplant recipients. The most frequent infections were complicated UTIs (nine [47%]), soft tissue infections (three [16%]), intra-abdominal infections (three [16%]) and catheter-related bacteraemia (two [11%]). The main characteristics and outcomes are summarised in Table 1, and Table 2 provides details for each patient.

Among the nineteen patients, nine VIM-type-producing and nine OXA-48-type-producing *Enterobacterales* and two VIM-type-producing *Pseudomonas aeruginosa* isolates were identified. One patient showed coinfection with two CP-GNB (patient 13). By disk diffusion, all *Enterobacterales* isolates were susceptible to ciprofloxacin, and all *P. aeruginosa* isolates were susceptible to increased exposure. The available in vitro antibiotic susceptibility pattern of all the 20 isolates is described in Table 3. Among the 11 available isolates, the median MIC of ciprofloxacin was 0.25 mg/L (IQR 0.125–0.35). Six (32%) patients had positive blood cultures, and one patient presented septic shock. The median duration of ciprofloxacin treatment was 14 days (IQR 10–15 days). Two patients received other active antibiotics (amikacin) during the first 24 h. Oral treatment was given to all but one patient, with a median duration of oral administration of 9 days (IQR 6–11 days). Six (32%) patients received only oral treatment.

One patient (5%), with OXA-48-producing *Enterobacter cloacae* bacteraemia due to a complicated UTI (acute pyelonephritis in a patient with a prior radical cystectomy), presented clinical failure at day 14 due to a lack of clinical improvement (patient 7). After 10 days of treatment with ciprofloxacin, the patient showed clinical worsening, with the appearance of focal pyelonephritis and bacteraemia due to ciprofloxacin-resistant ESBL-producing *E. coli*. There was no 30-day mortality. Furthermore, there were no relapsed infections at day 30, and there was one relapse at day 90: a ciprofloxacin-resistant OXA-48-producing *Klebsiella pneumoniae* in a catheter-related UTI (patient 5). There were two cases of microbiological recurrence at day 90. In addition to the patient who experienced clinical relapse, there was another case of microbiological recurrence at day 90: the isolation of ciprofloxacin-resistant VIM-producing *E. cloacae* from sputum, which was considered a colonisation (patient 9). In both cases of microbiological recurrence at day 90, the CP-GNB isolate was the same but developed resistance to ciprofloxacin (MIC > 32 mg/L). There were no reported adverse effects. 

## 3. Discussion

In this study, monotherapy with ciprofloxacin for non-severe CP-GNB infections resulted in favourable outcomes, with only one case of clinical failure at day 14 and no mortality at day 30. To the best of our knowledge, ciprofloxacin treatment of CP-GNB infections has not been published. Considering antibiotic stewardship, ciprofloxacin has several advantages. First, it does not have the broad antibiotic spectrum of the new beta-lactams. The use of ciprofloxacin could allow the sparing of new antibiotics, thereby reserving them for infections with no other active treatment [12]. In addition, fluoroquinolones have a high oral bioavailability, which facilitates early hospital discharge and reduces the side effects of intravenous catheters. In recent years, oral sequential therapy has been shown to be safe for other types of complex infections, such as *S. aureus* bacteraemia or endocarditis [13,14]. Oral treatment with fluoroquinolones has also shown its efficacy in osteoarticular infections [15]. Furthermore, oral fluoroquinolone treatment has also been successful in the step-down treatment of Enterobacterales bacteraemia [16,17]. Some limited data also showed similar results in AmpC- or ESBL-producing Enterobacterales infections treated with oral antibiotics, including fluoroquinolones [18,19]. Nevertheless, data on oral therapy for CP-GNB infections are scarce. Studies on other antibiotics with high bioavailability but also with high resistance rates, such as trimethoprim-sulfamethoxazole (SXT), have been reported [20,21]. In these studies, ten and twelve patients, respectively, with CP-GNB infections who were treated with SXT monotherapy achieved good clinical outcomes. The authors concluded that selected patients with non-severe CP-GNB infections could be treated with SXT [21]. It should be noted that in three/four cases with available follow-up cultures, resistance to SXT emerged [21]. In our study, microbiological recurrence was present in only two/nineteen patients (11%), but both isolates developed resistance to ciprofloxacin. The emergence of antibiotic resistance after treatment has also been described with other drugs [9,10,11,22], but should be considered, especially in cases of infection relapse. The most frequent ciprofloxacin resistance in these cases is due to gyrA and parC mutations [23,24]

In our study, six of the patients received only oral treatment. The spread of CP-GNB has meant that community-acquired infections are becoming more common [25,26]. In two multicentre studies, approximately 8% of CP-GNB infections were community-acquired, with UTIs being the most common infection [27,28]. Community-acquired CP-GNB infections are less severe and have a lower mortality, although active empiric treatment is less common [27]. We believe that our study is also important for reinforcing the safety of outpatient oral therapy for CP-GNB infections.

The main limitation to use ciprofloxacin in CP-GNB infections is the low susceptibility rate of these isolates. At our centre, the global susceptibility rate of CP-GNB to ciprofloxacin was 16%. These data are variable in the literature. According to a global analysis, the susceptibility rates of CP-GNB to ciprofloxacin are species-dependent, being 3% for *K. pneumoniae*, 8% for *E. coli* and 15% for *E. cloacae* [29]. In a more recent global epidemiological study including OXA-48 producing Enterobacterales, susceptibility to ciprofloxacin was also lower for *K. pneumoniae* (1.3%) than *E. coli* (8%) or *E. cloacae* (13%) [30]. However, susceptibility in Spain is slightly higher—7.6% in OXA-48-producing *K. pneumoniae* and 19% in carbapenemase-producing *E. coli* [31]. Finally, in China, 20.8% of ciprofloxacin susceptibility (MICs of ≤1 μg/mL) has been described in carbapenemase-producing *K. pneumoniae* [24].

Overall, this study has several limitations given its retrospective single-centre design and the absence of a control group. Furthermore, the identification of acquired resistance mechanisms to β-lactams, besides carbapenemases, was only phenotypically conducted. Consequently, it is possible that other β-lactamases were present but not detected. Nonetheless, this is a real-life experience describing ciprofloxacin treatment for CP-GNB infections.

## 4. Material and Methods

### 4.1. Patients and Settings

We performed an observational and retrospective study at Vall d’Hebron University Hospital, which is a 1000-bed tertiary teaching hospital in Barcelona, Spain. All ciprofloxacin-susceptible CP-GNB clinical isolates collected between January 2015 and July 2022 were retrospectively identified from the microbiology database. Consecutive adult patients (≥18 years of age) treated with ciprofloxacin monotherapy for at least 5 days were included. Patients with acute cystitis were excluded. Patients with infections for which another active antibiotic was used for >24 h were also excluded. The study protocol was approved by the hospital ethics committee EOM(AG)051/2022(6051)).

### 4.2. Outcomes and Definitions

Clinical and epidemiological variables of the patients were analysed. The Charlson index was used to assess the degree of comorbidity of the patients [32]. The glomerular filtration rate was calculated according to MDRD-4 [33]. Infections were defined according to the Centers for Disease Control and Prevention criteria (https://www.cdc.gov/nhsn/PDFs/pscManual/17pscNosInfDef_current.pdf, accessed on 1 February 2024) and were classified according to the type of acquisition [34]. Septic shock was defined with SEPSIS-3 criteria [35]. Ciprofloxacin dose, route of administration and total days of administration were collected.

Primary outcomes were clinical failure at day 14 and 30-day all-cause mortality from the index culture [36,37]. Secondary outcomes were infection relapse, microbiological recurrence and adverse reactions. We specifically searched for the presence of diarrhoea associated or not with *Clostridioides difficile*, tendinopathy or phototoxicity. Patients were followed up with for 90 days after finishing antibiotic treatment. Clinical failure was defined as lack of clinical improvement, a switch to another antibiotic because of lack of improvement or death during treatment. Infection relapse was defined as the occurrence of a second microbiologically documented CP-GNB infection with the return of clinical signs and symptoms of infection. Microbiological recurrence was defined as new CP-GNB isolation obtained after completing ciprofloxacin therapy when repeated cultures were available, regardless of the presence of signs or symptoms of infection. 

### 4.3. Microbiological Methods

Isolates included in this study were obtained from different types of samples that were collected, transported and cultured following standardised microbiological procedures. Bacterial isolates were identified by mass spectrometry (Vitek- MS; bioMérieux, Lyon, France). Antimicrobial susceptibility testing was performed by disk diffusion in all isolates. The MIC of ciprofloxacin was determined retrospectively by a gradient strip test (Etest; bioMérieux) for all isolates available in the clinical laboratory’s bacterial collection. Interpretation of the results was performed by applying the EUCAST clinical breakpoints available from 2015 to 2022. Carbapenemase production (KPC type, OXA-48 like, IMP type, VIM type and NDM type) was confirmed by lateral flow immunoassay (NG-Test Carba 5 assay, NG-Biotech, Guipry, France) according to the manufacturer’s recommendations.

### 4.4. Statistical Methods

The statistical package SPSS v24.0 (SPSS, Chicago, IL, USA) was used for data analysis. A descriptive analysis of the variables included in the study was carried out. Quantitative variables were described by median ± interquartile range. The qualitative variables were described by their absolute and relative frequencies.

## 5. Conclusions

In conclusion, monotherapy with ciprofloxacin could be an alternative treatment for selected, clinically stable patients with ciprofloxacin-susceptible GNB infections. In cases of relapse, the development of ciprofloxacin resistance must be considered.

## Figures and Tables

**Table 1:** Summary of clinical characteristics, microbiological isolations, treatments and outcomes of patients included.

	Patients (n = 19)
Age, years, median (IQR)	74 (66–79)
Sex, male, n (%)	15 (79%)
Charlson comorbidity index, median (IQR)	5 (3–6.5)
Immunocompromised, n (%)	8 (42%)
Kidney transplant recipients, n (%)	4 (21%)
Antibiotics prior 3 months, n (%)	15 (79%)
Hospitalisation prior 3 months, n (%)	12 (63%)
Length of hospital stay during infection episode, days, median (IQR)	37 (16–57.5)
ICU admission at infection onset, n (%)	4 (21%)
Type of infection, n (%)	
Nosocomial	14 (74%)
Healthcare-associated	5 (26%)
Community-acquired	0
Bacterial species, n = 20 (%)	
*Enterobacter cloacae* complex	9 (45%)
*Klebsiella pneumoniae*	4 (20%)
*Klebsiella oxytoca*	2 (10%)
*Pseudomonas aeruginosa*	2 (10%)
*Klebsiella aerogenes*	1 (5%)
*Serratia marcescens*	1 (5%)
*Escherichia coli*	1 (5%)
Carbapenemase class, n = 20 (%)	
OXA-48	9 (45%)
VIM	11 (55%)
Source of infection, n (%)	
Urinary	9 (47%)
Intraabdominal	3 (16%)
Skin and soft tissue	3 (16%)
Catheter-related bacteraemia	2 (11%)
Respiratory	1 (5%)
Primary bacteraemia	1 (5%)
Bacteraemia, n (%)	6 (32%)
Route of administration, n (%)	
Intravenous only	1 (5%)
Initial intravenous and oral sequential therapy	12 (63%)
Oral only	6 (32%)
Total CIP days, median (IQR)	10 (14–15)
Oral CIP days, median (IQR)	6 (9–12.5)
14 days failure, n (%)	1 (5%)
30 days mortality, n (%)	0
Infection relapse at day 90, n (%)	1 (5%)
Microbiological recurrence, n (%)	2 (11%)
Adverse effects, n (%)	0

Abbreviations: CIP: ciprofloxacin.

**Table 2:** Demographic, microbiological and clinical characteristics of 19 patients with carbapenemase-producing GNB treated with ciprofloxacin.

Patient	Age, Years (Sex)	Underlying Diseases	CCI	Type of Infection	Bacteraemia	Bacterial Species	Type of CP	MIC (mg/L) of CIP	IV CIP Therapy (Days) and Dose (eGFR)	Oral CIP Therapy (Days) and Dose (eGFR)	Clinical Failure at Day 14	Mortality at Day 30	Relapse at Day 90	Microbiological Recurrence at Day 90
1	66 (M)	SARS-CoV-2 pneumonia	3	Catheter-associated UTI	No	*P. aeruginosa*	VIM	0.125	Yes (4) ^a^, 400 mg/12 h (>90)	Yes (8), 750 mg/12 h (>90)	No	No	No	No
2	67 (M)	Kidney transplant recipient	5	Catheter-related bacteraemia	Yes	*E. cloacae*	VIM	0.5	No	Yes (8), 500 mg/12 h (28)	No	No	No	No
3	87 (F)	Cystocele, recurrent UTIs	2	Acute pyelonephritis	Yes	*E. cloacae*	VIM	NA	Yes (5) ^a^, 200 mg/12 h (41)	Yes (9), 500 mg/12 h (79)	No	No	No	No
4	78 (M)	Kidney transplant recipient	3	Acute graft pyelonephritis	No	*K. oxytoca*	VIM	0.25	No	Yes (12), 500 mg/12 h (38)	No	No	No	No
5	74 (M)	Radical cystectomy. B-cell-Lymphoma	7	Catheter-associated UTI	No	*K. pneumoniae*	OXA-48	0.047	No	Yes (7), 500 mg/12 h (>90)	No	No	Yes	Yes (CIP-resistant, MIC >32 mg/L)
6	58 (M)	Hip arthroplasty	2	Acute bacterial prostatitis	No	*E. cloacae*	VIM	0.25	No	Yes (14), 500 mg/12 h (89)	No	No	No	No
7	71 (F)	Radical cystectomy	5	Acute pyelonephritis	Yes	*E. cloacae*	OXA-48	NA	Yes (4), 200 mg/12 h (22)	Yes (5), 500 mg/12 h (58)	Yes ^b^	No	-	-
8	79 (M)	Chronic critical limb ischemia	8	Postoperative wound infection	No	*K. pneumoniae*	OXA-48	0.38	No	Yes (10), 500 mg/12 h (33)	No	No	No	No
9	86 (M)	Third-degree atrioventricular block	6	Tracheobronchitis	No	*E. cloacae*	VIM	0.25	Yes (4), 400 mg/12 h (29)	Yes (2), 500 mg/12 h (20)	No	No	No	Yes (CIP-resistant, MIC >32 mg/L)
10	73 (F)	Kidney transplant recipient	7	Acute graft pyelonephritis	No	*P. aeruginosa*	VIM	0.38	Yes (6), 200 mg/12 h (15)	Yes (8), 250 mg/12 h (18)	No	No	No	No
11	68 (M)	Oral squamous-cell carcinoma	9	Cervical postoperative collection	No	*K. pneumoniae*	OXA-48	NA	Yes (18), 400 mg/12 h (>90)	Yes (10), 750 mg/12 h (>90)	No	No	No	No
12	75 (M)	Small-cell lung cancer	9	Cholangitis	Yes	*E. coli*	OXA-48	NA	Yes (1), 400 mg/12 h (61)	Yes (13), 500 mg/12 h (61)	No	No	No	No
13	81 (F)	Femoro-femoral bypass graft	4	Postoperative wound infection	No	*E. cloacae* *K. pneumoniae*	OXA-48 OXA-48	0.016 NA	Yes (14) 400 mg/12 h (50)	No	No	No	No	No
14	54 (M)	Acute spinal cord injury	3	Catheter-related bacteraemia	Yes	*K. oxytoca*	VIM	NA	Yes (8), 400 mg/12 h (>90)	Yes (2), 750 mg/12 h (>90)	No	No	No	No
15	77 (M)	Pancreatic adenocarcinoma	5	Abdominal postoperative collection	No	*S. marcescens*	OXA-48	0.125	Yes (26), 400 mg/12 h (69)	Yes (5), 500 mg/12 h (75)	No	No	No	No
16	65 (M)	Intracerebral haemorrhage	5	Acute bacterial prostatitis	No	*E. hornaechei*	VIM	NA	Yes (8), 400 mg/8 h (>90)	Yes (14), 500 mg/12 h (>90)	No	No	No	No
17	75 (M)	Kidney transplant recipient	5	Peritonitis secondary to intestinal ischemia. Postsurgical collections	No	*E. cloacae*	VIM	NA	Yes (29), 200 mg/12 h (13)	Yes (29), 500 mg/12 h (43)	No	No	No	No
18	86 (M)	Heart failure, acute urinary retention requiring urethral catheter	2	Catheter-associated UTI	No	*K. aerogenes*	OXA-48	NA	No	Yes (10), 500 mg/12 h (52)	No	No	No	No
19	47 (M)	IMV due to drug-induced impaired consciousness	0	Unknown-origin bacteraemia	Yes	*E. cloacae*	VIM	0.38	Yes (2), 400 mg/8 h (>90)	Yes (14), 750 mg/12 h (>90)	No	No	No	No

Abbreviations: CCI, Charlson comorbidity index; CIP, ciprofloxacin; CP, carbapenemase; eGFR, estimated glomerular filtration rate (mL/min/1.73 m^2^); F, female; h, hours; IMV, invasive mechanical ventilation; IV, intravenous, M, male; MIC, minimum inhibitory concentration; NA, not available; SARS-CoV-2, severe acute respiratory coronavirus 2; UTI, urinary tract infection. ^a^ These patients also received one dose of intravenous amikacin during the first 24 h. ^b^ This patient presented clinical worsening, with the appearance of a perirenal abscess and bacteraemia due to ciprofloxacin-resistant extended-spectrum β-lactamase-producing *E. coli*. She was successfully treated with ceftazidime/avibactam for 30 days.

**Table 3:** In vitro antibiotic susceptibility pattern of 20 isolates from 19 patients with carbapenemase-producing Gram-negative bacteria infections treated with ciprofloxacin.

Patient	Bacterial Species	Acquired Beta-Lactamases	CAZ	IPM	MEM	ETP	GEN	AMK	CIP	SXT
1	*P. aeruginosa*	VIM	R	R	R	NA	S	S	S	NAp
2	*E. cloacae*	VIM	R	R	I	R	R	S	S	R
3	*E. cloacae*	VIM	R	I	S	R	R	S	S	R
4	*K. oxytoca*	VIM	R	R	NA	S	R	S	S	R
5	*K. pneumoniae*	OXA-48 + ESBL	R	R	NA	R	S	NA	S	S
6	*E. cloacae*	VIM	R	S	S	R	R	S	S	S
7	*E. cloacae*	OXA-48	R	S	NA	R	S	NA	S	S
8	*K. pneumoniae*	OXA-48	S	S	S	R	S	S	S	S
9	*E. cloacae*	VIM	R	R	R	R	R	S	S	S
10	*P. aeruginosa*	VIM	R	R	R	NA	R	S	S	NAp
11	*K. pneumoniae*	OXA-48	R	R	I	R	S	S	S	S
12	*E. coli*	OXA-48	S	S	NA	I	S	S	S	S
13	*E. cloacae*	OXA-48	R	R	I	R	S	S	S	R
	*K. pneumoniae*	OXA-48	R	R	R	R	R	S	S	R
14	*K. oxytoca*	VIM	R	I	I	R	R	I	S	S
15	*S. marcescens*	OXA-48	R	R	NA	R	S	S	S	S
16	*E. hornaechei*	VIM	R	R	I	R	R	NA	S	R
17	*E. cloacae*	VIM + ESBL	R	I	R	R	I	S	S	R
18	*K. aerogenes*	OXA-48	R	R	R	R	S	S	S	S
19	*E. cloacae*	VIM	R	S	S	S	R	S	S	S
**Total susceptibility (%) (including S and I)**			10	40	60	16	50	100	100	61

AMK: amikacin; CAZ: ceftazidime; CIP: ciprofloxacin; ETP: ertapenem; GEN: gentamicin; I: susceptible increased exposure; IPM: imipenem; MEM: meropenem; NA: not available; NAp: not applicable; R: resistant; S: susceptible; SXT: trimethoprim-sulfamethoxazole.

## Data Availability

The datasets presented in this article are not readily available because we do not have permission from the ethical committee.
